# Athletes Drive Distinctive Trends of COVID-19 Infection in a College Campus Environment

**DOI:** 10.3390/ijerph18147689

**Published:** 2021-07-20

**Authors:** Austin T. Hertel, Madison M. Heeter, Olivia M. Wirfel, Mara J. Bestram, Steven A. Mauro

**Affiliations:** Department of Biology, Morosky College of Health Professions and Sciences, Gannon University, Erie, PA 16541, USA; hertel002@gannon.edu (A.T.H.); heeter004@gannon.edu (M.M.H.); wirfel002@gannon.edu (O.M.W.); bestram001@gannon.edu (M.J.B.)

**Keywords:** COVID-19, SARS-CoV-2, RT-PCR, university, athletes

## Abstract

The COVID-19 pandemic forced most institutions of higher education to offer instruction and activities offsite, impacting millions of people. As universities consider resuming normal operations on campus, evidence-based guidance is needed to enhance safety protocols to reduce the spread of infectious disease in their campus environments. During the 2020/2021 academic year, Gannon University in Erie, PA, USA, was able to maintain most of its operations on campus. Part of Gannon’s disease mitigation strategy involved the development of a novel in-house, real-time RT-PCR-based surveillance program, which tested 23,227 samples to monitor the presence of COVID-19 on campus. Temporal trends of COVID-19 infection at Gannon were distinct from statewide data. A significant portion of this variance involved student athletes and associated staff, which identified as a higher incidence risk group compared with non-athletes. Rapid identification of athlete driven outbreaks allowed for swift action to limit the spread of COVID-19 among teammates and to the rest of the campus community. This allowed for successful completion of instruction and a modified season for all sports at Gannon. Our findings provide insights that could prove useful to the thousands of institutions seeking to resume a more traditional presence on campus.

## 1. Introduction

The COVID-19 pandemic severely disrupted higher education, displacing millions of students and personnel from the thousands of institutions of higher learning globally. In the fall of 2020, of 905 international universities outside the United States, only 7% offered classes primarily in-person, which is defined as classes conducted in person with some exceptions for online delivery. Of 1442 institutions of higher education in the United States during this same timeframe, only 3% offered fully in-person instruction. For the 97% of institutions that did not resume normal operations, 28% offered classes primarily in person, while another 22% offered instruction in a hybrid model, which is classified as some weeks/days online and some weeks/days in person. Another 31% taught primarily or fully online [1].

For institutions that remained open to students, campus activities such as athletics presented significant challenges. Athletes make up a significant portion of the campus population of the 1098 institutions that have membership with one of the three divisions of the NCAA. More specifically, 1 in 23 students (4%) are athletes for NCAA Division 1 affiliates, 1 in 10 (10%) for Division 2, and 1 in 6 (17%) for Division 3 [2]. In total, this accounts for hundreds of thousands of student-athletes across the United States. Given these numbers, the threat of COVID-19 outbreaks within this population is a possibility. In support of this, one such report demonstrated that repeat social gatherings with limited preventative measures (mask use and social distancing), led to 17 confirmed cases of COVID-19 within a university soccer team [3].

Additional outbreaks of COVID-19 within a university environment have been characterized. In August of 2020, a university in North Carolina recorded 670 new cases of COVID-19 within 22 days of reopening [4], resulting in the university transitioning all classes to online format. Similarly, an outbreak on a university campus in Indiana resulted in 371 new cases between 16–22 August 2020 [5]. This prompted the university to transition classes online on 19 August 2020 before returning to in-person instruction on 2 September 2020. Additionally, Duke University identified 84 new cases from 2 August 2020–11 October 2020, which did not require a transition to online classes [6]. While these studies present responses to individual outbreaks, a comprehensive analysis of COVID-19 management strategies spanning an entire academic year has not been reported. Such evidence-based guidance is needed as easing public restrictions and increased vaccine availability are leading universities to consider resuming fully in-person operations. This is especially true since SARS-CoV-2 variants among other emerging pathogens could leave institutions vulnerable to outbreaks.

In response to the pandemic, Gannon University implemented significant operational changes during the 2020–2021 academic year to maintain a level of on campus presence for students and staff. Gannon is a small, private university with 4755 students and personnel on its campus in Erie, Pennsylvania. There are 3208 students enrolled in undergraduate programs, including 705 student-athletes and associated staff. Gannon offers an array of athletic programs, including those that are classified by NCAA guidelines as high, intermediate, and low risk for the transmission of COVID-19 [7].

One of the safety measures Gannon required on campus included gathering limitations to reflect local COVID-19 guidelines, along with mask requirements at all times, unless eating. To allow for social distancing in the classroom, technology upgrades for in-person classes were installed to allow for simultaneous remote and in-person lecture delivery when necessary. Temperature check stations were utilized before entering high traffic buildings, and room sanitation was conducted before the start of each class. Gannon also made use of a mobile app that required students and personnel to complete a daily symptom survey. If an individual reported COVID-19 symptoms, they would be flagged and contacted by university health staff to schedule a COVID-19 test.

A unique feature of Gannon’s COVID-19 response included an in-house real-time RT-PCR based surveillance program, which allowed for regular daily testing and delivered results within 8–12 h from the time samples were taken. The results obtained from the 23,227 samples analyzed throughout the academic year made it possible for rapid contact tracing, isolation, and quarantine responses, also facilitated by Gannon employees. These practices made it possible for Gannon to offer 903 of its 1221 classes (75%) fully in-person in the 2020 fall semester. Of the remaining 25% of classes, 249 (20%) utilized a hybrid model, and only 69 classes (5%) were taught remotely. In addition, Gannon was able to facilitate activity and competition for all athletic teams in accordance with NCAA guidance for the entire academic year.

Despite stringent mitigation strategies, Gannon University was not without outbreaks of COVID-19 on its campus environment, many of which were attributed to the student-athlete population. However, with rapid response and continued surveillance, Gannon was able to maintain classroom instruction as designed, without interruption for both the 2020 fall and 2021 spring semesters. Additionally, all sports were able to complete a modified season for the duration of the academic year. The results of our year-long COVID-19 surveillance program provide insights that may prove useful to the thousands of institutions seeking to resume a more traditional presence, while limiting the spread of COVID-19 and other infectious diseases on their campus environment.

## 2. Materials and Methods

### 2.1. Sample Classification

Classification of student and employee groups for analysis were determined at the time of test scheduling and confirmed during sample collection. For athletes, group size includes all student-athletes and coaching staff on the roster during the 2020/2021 academic year. Transmission risk was classified according to NCAA guidelines, which is based on sport-specific environments that would contribute to the likelihood of the spread of respiratory droplets during vigorous activity. Common characteristics of sports classified as high risk for transmission of COVID-19, were sports played indoors (or in poorly ventilated spaces) and/or environments where sport-specific activities demand frequent close contact between competitors and teammates. Low/intermediate risk sports were characterized by outdoor environments where physical distancing can be consistently maintained and/or by sport-specific activities that facilitated at most, short-lived close contact between teammates and competitors. One exception of our classification to that of the NCAA guidelines was water sports. Swimming and diving, which is listed by the NCAA guidelines as low risk, was combined with the high-risk water polo because of the overlap of these student-athletes between these three sports.

The non-athlete group size included all Erie campus students and employees minus the total athlete population. Randomized testing, or general surveillance, of select non-athlete groups included students in classes, labs, on clinical rotation, or in dorm rooms or other communal living situations like fraternities or sororities. A health center visit refers to individuals who were directed to the health center because of a failed symptom check, who otherwise reported symptoms, or who had known exposure to someone infected with SARS-CoV-2. All personal information collected received IRB approval and were anonymized prior to receiving and processing in the lab to comply with Health Insurance Portability and Accountability Act of 1996 (HIPAA) and privacy standards.

### 2.2. Sample Collection and Preparation

Nasopharyngeal specimens were obtained by trained university health staff observing standard procedures and stored in 2 mL of viral transport media (VTM) containing 1X Hanks Balanced Salt Solution, 2% Fetal Bovine Serum, 0.1 mg/mL gentamicin, and 0.5 µg/mL amphotericin B. All samples were transported on ice in secondary containers to the testing laboratory and processed within a four-hour timeframe. A modified version of the CDC Emergency Use Authorization (EUA) real-time RT-PCR protocol [8] was used to detect SARS-CoV-2 in specimens using the Applied Biosystems 7500 platform (Waltham, MA, USA). Specimens were resuspended in VTM by vortexing for 10 sec and then transferring 500 µL to an Eppendorf tube. Boiling samples at 368.15 K for 5 min was utilized to extract RNA from specimens [9].

### 2.3. Real-Time RT-PCR

#### 2.3.1. Operating Procedure

For real-time RT-PCR, we added 5 µL of resuspended sample to 15 µL of PCR mixture containing 1X Applied Biosystems TaqPath 1-Step RT-qPCR Master Mix (Waltham, MA, USA) and the primer/probe mix targeting the N1 gene of SARS-CoV-2. The primers and probes were available from IDT Integrated DNA Technologies (Coralville, IA, USA) and were used at a final concentration of 2.5 µM forward primer (nCOV_N1 Forward Primer, 5′-GACCCCAAAATCAGCGAAAT-3′), 2.5 µM reverse primer (nCOV_N1 Reverse Primer, 5′-TCTGGTTACTGCCAGTTGAATCTG-3′), and 2.5 µM probe (nCOV_N1 Probe, 5′-FAM-ACCCCGCATTACGTTTGGTGGACC-BHQ1-3′). The reactions were performed using an Applied Biosystem 7500 platform with a thermal cycle of one repetition each at 298.15 K for 2 min, 323.15 K for 15 min, and 368.15 K for 2 min. To amplify the genetic material, the temperature was cycled from 368.15 K for 3 s followed by 328.15 K for 30 s for a total of 45 repetitions.

#### 2.3.2. Interpretation of Results

Results of the real-time RT-PCR protocol were compared with a standard curve generated from results using the 2019-nCoV_N_Positive Control plasmid from IDT Integrated DNA Technologies (Coralville, IA, USA). A result was classified as positive if it fell within the linear range of amplification of the standard control curve. We used 1000 copies as the lower limit of detection, which is consistent with what has been reported [10]. All individuals that tested positive for the presence of SARS-CoV-2 using these parameters were scheduled for a follow-up diagnostic test within 24 hours for confirmation in Clinical Laboratory Improvement Amendments (CLIA) certified, independent laboratories.

## 3. Results

### 3.1. Gannon University Real-Time RT-PCR Surveillance

Between 24 August 2020 and 12 May 2021, Gannon’s surveillance program completed 23,227 tests, which identified 235 confirmed cases (1.01%) of COVID-19. Each positive result by Gannon was sent for follow-up, confirmatory testing to compare the results of our testing procedure with that of an independent CLIA certified laboratory. Of the 235 samples that tested positive for the presence of SARS-CoV-2 by Gannon, 98.7% were consistent with follow-up testing (Table 1). In rare cases, we detected a signal by real-time RT-PCR that was below our established limit of detection and was considered a negative test result. A subset of these samples was also tested in an independent laboratory, which produced results that were consistent with ours 85% of the time. To further compare our negative results that did not produce any signal by real-time RT-PCR, 146 samples were sent for follow-up diagnostic testing in an independent lab. The results from this comparison demonstrated 100% consistency between laboratories.

### 3.2. Temporal Patterns of COVID-19 Infection

**Figure 1:**
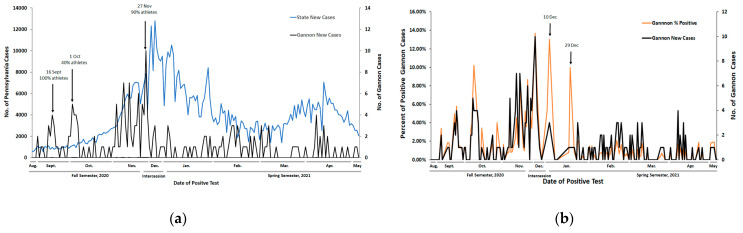
Temporal trends of COVID-19 infection at Gannon University and Pennsylvania, United States. Data points reflect only days between the time periods indicated that Gannon University processed samples. n = 179 total days of sampling. (**a**) Cumulative number of daily samples testing positive for COVID-19 infection between 24 August 2020–12 May 2021; (**b**) percent of samples testing positive for COVID-19 infection between 24 August 2020–12 May 2021.

Comparison of Gannon temporal trends of new SARS-CoV-2 infections to Pennsylvania new cases indicated patterns that were distinctive from state-wide data (Figure 1a). Pennsylvania state exhibited a gradual increase in the number of COVID-19 cases between 3 December 2020 and 28 January 2021, recording its largest count of 12,798 new cases on 10 December 2020. An additional increase in state cases was identified between 19 March 2021 and 29 April 2021, peaking at 7078 cases on 12 April 2021. In contrast, Gannon experienced episodic rises in cases including two small outbreaks occurring 40 days into the fall semester—the first on 16 September 2020 (four positive cases) and again on 1 October 2020 (five positive cases). Gannon’s highest daily case count of COVID-19 occurred on 27 November 2020 when it recorded 10 new cases. Several of Gannon’s outbreaks that were distinguishable from state trends were marked with a high percentage of infection among student-athletes. For example, 90% of positive cases recorded on 27 November 2020 were student-athletes. Additionally, student-athletes made up 40% of all cases on 1 October 2020, and 100% of all cases on 16 September 2020.

To gain insight into how Gannon’s daily new cases related to testing volume, we compared the percent of daily samples positive for COVID-19 to total new cases of COVID-19 per day at Gannon. (Figure 1b). While the trends between the two curves were similar, there were some occurrences when the percent of samples positive for COVID-19 at Gannon did not align with the number of new cases. For example, a high percentage of samples positive for COVID-19 (13.0%) at Gannon was recorded on 10 December 2020, which was not reflected by a high number of new cases on that day (Figure 1b). This is also true of the high percent positivity of new COVID-19 cases recorded on 29 December 2020 (10.0%) compared with the relatively low number of new cases recorded on that day. With these exceptions, the trends between new COVID-19 cases at Gannon and the percentage of samples positive for COVID-19 were similar. This is also reflected when comparing the total case counts with the percent of samples positive for COVID-19 for those tested statewide (Figure S1).

### 3.3. SARS-CoV-2 Surveillance of Athlete and Non-Athlete Groups

To better understand the relationship of COVID-19 infection patterns between athletic teams and non-athletes, a closer examination of infection within these distinct populations was conducted (Table 2). The percent of samples positive for COVID-19 between individual sports was comparable regardless of risk classification (*p* = 0.26 between high risk and low/intermediate risk athlete populations). For example, the percent of samples positive for COVID-19 taken from wrestlers, which has a high risk of COVID-19 transmission, was 0.66%, which is similar to the 0.60% of samples positive for COVID-19 taken from baseball players, which is a low/intermediate risk of COVID-19 transmission sport. Additionally, we found these results were comparable to the general surveillance of the non-athlete population (all below 1.5%). However, the percent of samples positive for COVID-19 for non-athletes that were direct visits to the health center was considerably higher (6.78%). Direct health center visits were typically symptomatic individuals or those who were known close contacts.

### 3.4. Incidences of COVID-19 Infection

**Figure 2:**
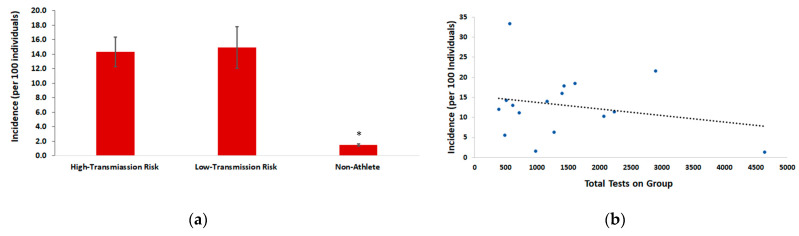
Incidence of COVID-19 infection of high-transmission risk athletes, low-transmission risk athletes, and non-athletes during the 2020/2021 academic year at Gannon University, Erie campus. (**a**) Incidence of COVID-19 infection of athlete group (high-transmission risk or low-transmission risk as classified in Table 2) and non-athletes. Values represent average incidence per 100 individuals of groups represented in Table 2. Error bars are the standard error. *Denotes a *p*-value below 0.02 using a two-tailed Student’s t-test when comparing average value of non-athletes to high-risk or low-risk of COVID-19 transmission athletes. (**b**) Linear regression of average incidence of COVID-19 infection for groups represented in Table 2 compared with total COVID-19 tests performed on that group.

While student-athlete groups showed relatively low percent of samples that were positive for COVID-19, they constituted 103 of the 224 cases of COVID-19 on days where athlete status was known, emphasizing their importance as a major reservoir for this virus at Gannon. The average incidence of COVID-19 in athletic teams and staff was 14.7, and ranged from 5.6 to as high as 33.3, all markedly higher than the incidence observed in non-athletes. Figure 2a elucidates the incidence of COVID-19 between athlete and non-athlete groups. Both high risk (14.3) and low/intermediate risk (14.9) transmission groups experienced greater average incidence than non-athletes (1.50). The incidence of the low/intermediate risk athlete group was comparable to the high-risk athlete group (*p*-value greater than 0.05 when comparing average incidences between these two groups).

We noted that the total number of tests administered between different groups varied during the study period, with a particular bias towards athletes. To understand if differences in volume of testing between groups could be driving the incidence rates, testing data from several non-athlete groups that were surveilled with increased frequency were further analyzed. Five non-athlete groups (fraternities, sororities, extracurricular clubs, etc.) of comparable size to athletic teams were scheduled for repeat surveillance testing between five and six occasions. For each of the days where surveillance testing of these non-athlete groups occurred, there were no new cases of COVID-19 identified. In comparison, the average incidence of COVID-19 within athletic teams and staff was 14.7, which is strikingly higher than any non-athlete group subject to repeat surveillance.

To further analyze the impact of sampling number on incidence rate, we plotted the number of tests performed for each cohort in relationship to the incidence of COVID-19 (Figure 2b). We found that there was no correlation (R^2^ = 0.052) between the number of tests performed and the incidence of COVID-19 for the groups that we tested (Figure 2b). We also analyzed by linear regression the incidence of individual athletic teams in relation to the approximate frequency (in days) in which they were tested. Here, we also found no significant correlation (R^2^ = 0.048) between the frequency of testing days and the incidence of COVID-19 in teams.

## 4. Discussion

To meet the need of high-throughput COVID-19 testing at Gannon’s campus, we developed a modified, but novel in-house surveillance strategy which allowed for rapid and regular COVID-19 screening of students and personnel for the entire academic year. By comparing the results of our findings with an independent CLIA certified reference lab (Table 1), we show that our approach is effective in identifying cases of COVID-19 in our campus environment. More specifically, samples that were designated as positive were in alignment with the reference laboratory 98.7% of the time. Samples that did not have a signal by real-time RT-PCR and were designated as negative, were in alignment with the reference laboratory 100% of the time. It is noteworthy that in some cases a signal was detected by real-time RT-PCR but was below our reliable limit of detection, which we classified as negative. There were three instances of results of this nature that were designated as positive by follow-up testing in a reference laboratory, indicating a discrepancy between the two labs. This suggests that some positive specimens may not have viral loads which can be reliably distinguished by this limit of detection. Additionally, the timing of testing can influence the detectable signal produced by presence of SARS-CoV-2 in an individual, with a mean incubation period of 4–5 days post-exposure [11]. This implies that tests administered to infected individuals before this period may not have sufficient viral load to produce detectable signal. Lowering the minimum threshold of detection is a strategy that can increase the likelihood of identifying samples with low SARS-CoV-2 viral load but risks an increased number of false positive results. Regardless, our results were remarkably consistent with those of a CLIA certified reference lab and allowed for a rapid and aggressive public health response to outbreaks of COVID-19 on campus. Hence, while we were unable to identify all COVID-19 cases within our campus environment, our strategy was effective in mitigating COVID-19 spread in a way that allowed Gannon to resume in-person instruction and activities for the entire academic year.

The low number of COVID-19 cases at Gannon suggests that the strategy of preventative measures (e.g., masks, social distancing, and frequent sanitation) in combination with regular testing [12] has contributed to Gannon’s ability to maintain in-person operations. It is noteworthy that there is a distinct temporal pattern of new COVID-19 cases on Gannon’s Erie campus compared with statewide data (Figure 1a). For most days, the trend of percent of samples positive for COVID-19 closely reflected the new case count, indicating that spikes in cases were not driven by changes in the volume of testing (Figure 1b). The few exceptions were on days where total volume of testing was low.

These results demonstrate low, but persistent presence of COVID-19 infection on Gannon’s campus that is distinctive of statewide trends, which has two important implications. First, despite adoption of national and state guidance for mitigating spread of COVID-19 on campus, along with enhancing measures that included rapid surveillance testing, Gannon was only able to limit but not eliminate the occurrence of outbreaks of COVID-19 on its campus. This suggests that state guidance and enhanced protocols are necessary but not sufficient in mitigating the spread of COVID-19 in a university campus environment. Second, our data provides evidence that the unique trends we observe at Gannon compared with statewide data are not largely due to difference in daily testing volume, but instead arise from unique features of the campus community which cannot be easily predicted from statewide trends.

One of the distinguishing populations responsible for outbreaks of COVID-19 on our campus are student-athletes (Figure 1, Table 1, Figure 2). For days where athlete status was known, 45.9% of all COVID-19 cases on Gannon’s campus were student-athletes (Table 2). An important note is that athletes across risk designations experienced a comparable percent of samples positive for COVID-19. Athletic teams designated as high-risk for transmission of COVID-19 had an average incidence of 14.3, which is remarkably similar to the average incidence of low/intermediate risk athletic teams of 14.9. This indicates that student-athletes as a population, regardless of the risk designation for individual sports are prone to contracting COVID-19, and should be surveilled in increased, and comparable volumes across all sports. This demonstrates that risk classifications for sports is not a relevant indicator for transmission risk of COVID-19. A plausible explanation for this finding is that strict preventative measures (masking, temperature checks, frequent sanitation, and symptom reporting, etc.) are in place during practices and competition for all sports at Gannon, which may decrease the risk of transmission irrespective of risk factors. Regardless, given the high incidence of COVID-19 found in athlete groups within our population, studies that further explore events and/or behaviors associated with the transmission of COVID-19 are warranted.

The percent of samples from student-athletes that tested positive for COVID-19 was relatively low, indicating that testing volume was efficient in identifying cases within this group. Despite this, we noted disproportionately high COVID-19 incidence within this population compared with non-athletes (Figure 2). The risk ratio associated with these incidences demonstrates that student-athletes on Gannon’s campus were nearly 5 times more likely to contract COVID-19 compared with non-athletes. While the exact reason for higher incidence of COVID-19 in student-athletes is unknown, the information gathered from contact tracing suggests repeated group gatherings, that are non-compliant with safety protocols, rather than sport related activities are likely reasons. A possible explanation is that athletes in university environments typically coexist in larger social groups than other campus populations and frequently interact with other team members, and across athletic teams. Frequent and sustained social interactions present a large risk for the spread of contagion [13], which is evidence to suggest that athletes as a university population are at high risk for contracting COVID-19. In support of this, an outbreak of COVID-19 on a university men’s and women’s soccer team demonstrated by whole-genome sequencing that 17 new cases across both teams, likely emanated from a single introduction of SARS-CoV-2 during multiple social gatherings between teams, where preventative measures were not taken [3].

An alternative explanation for the higher incidence of COVID-19 infection in athletes is sampling bias, given that student-athletes were tested with greater frequency than non-athletes. However, three pieces of evidence suggest that this is not the case. First, we found no correlation between the number of tests performed on a group and the incidence of infection. Second, the frequency in which a particular athlete group was tested showed no statistical relationship between COVID-19 incidence rates. Lastly, of the non-athlete groups that were tested repeatedly (five times or greater) during the study period, there was not a single case of COVID-19 identified on days these groups were selected for surveillance testing.

Together, this information provides strong evidence that sampling frequency is not a major contributing factor to the high incidence of COVID-19 observed in athletes compared with the general student population. These data were an important factor in the decision to allocate our focus and resources on efforts that increase rapid surveillance testing of student-athletes, allowing us to make critical public health decisions with limited resources. However, we cannot entirely exclude the possibility that increasing the sampling frequency of other non-athlete populations could reveal a high COVID-19 incidence in our other campus environments, which has been documented at other institutions [4,5,6]. Thus, if resources allow, more frequent surveillance of all campus populations would be the optimal approach for fully understanding COVID-19 transmission patterns in all campus environments.

COVID-19 outbreaks are characteristic of university populations, as reported at other institutions across the United States [3,4,5,6]. This article is unique in that it follows patterns of COVID-19 infection over an entire academic year. The importance of identifying effective mitigation strategies on university campuses to reduce transmission to individuals at greatest risk of severe complications has been demonstrated [6,14]. Successful COVID-19 responses to outbreaks in campus environments have utilized strategies which identified symptomatic individuals by routine symptom monitoring, in combination with random surveillance testing of the general population, and routine testing of select populations [5,6]. Our results provide insights into factors that may be responsible for the distinctive outbreak patterns characteristic of campus environments. Student-athletes have been demonstrated to be at increased risk for contracting COVID-19 in this setting.

A limitation of this study is that the trends observed at Gannon’s Erie campus may not be generalizable to other institutions. This might especially become true given that COVID-19 vaccinations are becoming more readily available. However, the emergence of new SARS-CoV-2 variants and other outbreaks of infectious disease on college campuses remains threatening. Given the millions of students attending college globally, many of which are intending to resume athletic participation, the recommendation to monitor the spread of COVID-19, particularly within the student-athlete population, should be strongly considered. Additional studies are needed to further establish the specific role student-athletes have in facilitating the spread of COVID-19 within a campus environment.

## 5. Conclusions

Our results demonstrate that student-athletes are a major driver of COVID-19 infection in a university campus environment. The rapid identification of athlete driven outbreaks by an in-house real-time RT-PCR testing procedure allowed for swift action of any positive cases to limit the spread of COVID-19 among teammates and to the rest of the campus community. However, student-athletes still experienced a much higher incidence of COVID-19 than the rest of the campus population. Based on this information, a focus on enhanced surveillance and prevention strategies which targets the entire student-athlete population is warranted. In addition, since our study could not fully exclude the possibility that other student populations are prone to high COVID-19 incidence rates, routine testing of all student populations is recommended if resources are available. Adoption of these procedures will likely facilitate successful reopening strategies and athletic events for institutions seeking a more traditional presence on campus.

## Figures and Tables

**Table 1:** Results comparing the total number and percent of results of Gannon COVID-19 testing to that of an independent CLIA certified reference laboratory.

Comparison to Reference Laboratory	Positive Samples (≥1000 Copies) ^1^	Negative Samples (<1000 Copies) ^2^	Negative Samples (No Signal)
No (%) results matching	232 (98.7%)	17 (85.0%)	146 (100.0%)
No (%) results non-matching	3 (1.3%)	3 (15.0%)	0 (0.0%)

^1^ Copies denote the normalization of signal to a standard curve as described in the Materials and Methods section. ^2^ Those instances where signal was detected but were below the reliable established threshold were characterized as negative for COVID-19.

**Table 2:** Characteristics of SARS-CoV-2 surveillance of athlete and non-athlete group types at Gannon University, Erie, Pennsylvania Campus, during the 2020/2021 academic year.

Group Type	Group Size	No. Tests ^1^	No. (%) Positive Tests
**High Risk Athlete**			
Acrobatics/Tumbling	48	1271	3 (0.24%)
Basketball	38	1607	7 (0.44%)
Competitive Cheer/Dance	67	1430	12 (0.84%)
Football	96	2235	11 (0.49%)
Water Sports	88	2068	9 (0.44%)
Wrestling	88	2895	19 (0.66%)
**Low/Intermediate Risk Athlete**			
Athletic Staff	18	482	1 (0.21%)
Baseball	50	1161	7 (0.60%)
Cross Country	35	509	5 (0.98%)
Golf	25	387	3 (0.78%)
Lacrosse	23	607	3 (0.49%)
Soccer	81	1401	13 (0.93%)
Softball	27	713	3 (0.42%)
Volleyball	21	567	7 (1.23%)
**Non-Athlete**			
Health Center	4035	974	66 (6.78%)
General Surveillance	4035	4642	55 (1.18%)

^1^ The total number of tests is the cumulative sum of all tests taken on all individuals within a group type over the course of the 2020/2021 academic year. Some individuals may have been tested on multiple occasions. Athlete status was not known on seven testing days. These results were excluded from this analysis.

## Data Availability

Data presented in this study are available upon request from the corresponding author.

## Supplementary Materials

The following are available online at https://www.mdpi.com/article/10.3390/ijerph18147689/s1, Figure S1: Temporal trends of percent and total COVID-19 infection at Gannon in Pennsylvania, United States.

## References

[1] The College Crisis Initiative (C2i). https://collegecrisis.shinyapps.io/dashboard/.

[2] National Collegiate Athletic Association Our Three Divisions. https://res.mdpi.com/data/mdpi_references_guide_v5.pdf.

[3] Teran R.A., Ghinai I., Gretsch S., Cable T., Black S.R., Green S.J., Perez O., Chlipala G.E., Maienschein-Cline M., Kunstman K.J. (2020). COVID-19 Outbreak among a University Men’s and Women’s Soccer Teams—Chicago, Illinois, July–August 2020. MMWR Morb. Mortal. Wkly. Rep..

[4] Wilson E., Donovan C.V., Campbell M., Chai T., Pittman K., Seña A.C., Pettifor A., Weber D.J., Mallick A., Cope A. (2020). Multiple COVID-19 Clusters on a University Campus—North Carolina, August 2020. MMWR Morb. Mortal. Wkly. Rep..

[5] Fox M.D., Bailey D.C., Seamon M.D., Miranda M.L. (2021). Response to a COVID-19 Outbreak on a University Campus—Indiana, August 2020. MMWR Morb. Mortal. Wkly. Rep..

[6] Denny T.N., Andrews L., Bonsignori M., Cavanaugh K., Datto M.B., Deckard A., DeMarco T.C., De Naeyer N., Epling C.A., Gurley T. (2020). Implementation of a Pooled Surveillance Testing Program for Asymptomatic SARS-CoV-2 Infections on a College Campus—Duke University, Durham, North Carolina, August 2–October 11, 2020. MMWR Morb. Mortal. Wkly. Rep..

[7] National Collegiate Athletic Association Resocialization of Collegiate Sport: Developing Standards for Practice and Competition Guidelines. https://ncaaorg.s3.amazonaws.com/ssi/COVID/SSI_ResocializationDevelopingStandardsSecondEdition.pdf.

[8] Centers for Disease Control and Prevention CDC 2019-Novel Coronavirus (2019-nCoV) Real-Time RT-PCR Diagnostic Panel. https://www.fda.gov/media/134922/download.

[9] Beltran P.C., Alonso-Palomares L.A., Valiente-Echeverria F., Gaggero A., Soto-Rifo R., Barriga G.P. (2021). Accuracy of a RT-qPCR SARS-CoV-2 detection assay without prior RNA extraction. J. Virol. Methods.

[10] Wölfel R., Corman V.M., Wolfgang G., Seilmaier M., Sabine Z., Müller M.A., Niemeyer D., Kelly T.C.J., Vollmar P., Rothe C. (2020). Virological assessment of hospitalized cases of coronavirus disease 2019. medRxiv.

[11] Centers for Disease Control and Prevention Overview of Testing for SARS-CoV-2 (COVID-19). https://www.cdc.gov/coronavirus/2019-ncov/hcp/testing-overview.html.

[12] Manabe Y.C., Sharfstein J.S., Katrina Armstrong K. (2020). The Need for More and Better Testing for COVID-19. JAMA.

[13] Scala A., Flori A., Spelta A., Brugnoli E., Cinelli M., Quattrociocchi W., Pammolli F. (2020). Time, space and social interactions: Exit mechanisms for the Covid-19 epidemics. Sci. Rep..

[14] Walke H.T., Honein M.A., Redfield R.R. (2020). Preventing and Responding to COVID-19 on College Campuses. JAMA.

