# Supplementary material for: Athletes Drive Distinctive Trends of COVID-19 Infection in a College Campus Environment
Source: Int J Environ Res Public Health. 2021 Jul 20;18(14):7689. doi: 10.3390/ijerph18147689 (PMC8307320; doi:10.3390/ijerph18147689)
Supplement: Supplementary file 1 [file ijerph-18-07689-s001.zip › ijerph-1277432-supplementary.pdf]

Supplementary Material

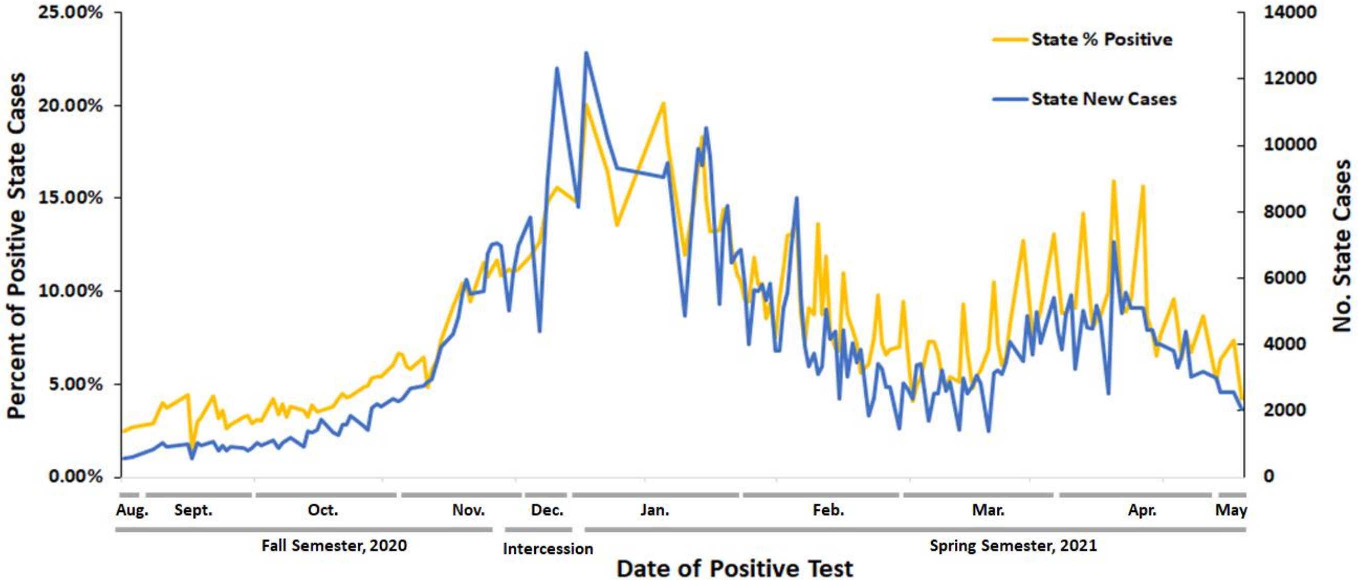

Figure S1. Temporal trends of percent and total COVID-19 infection at Gannon in Pennsylvania, United States.
